# Security from Yellow Fever

**Published:** 1888-10

**Authors:** 


### Editorial.

### SECURITY FROM YELLOW FEVER.

Daniel's *Texas Medical Journal* has seen fit to take us to task for our editorial in the September number upon yellow fever, and to accuse us of boasting of an immunity from danger of infection in Atlanta without sufficient grounds. Referring to our statement that "the history of the disease in the past has shown that no city of the altitude of Atlanta has ever been ravaged by yellow fever," our contemporary is "somewhat surprised at our (his) brother's lack of information on the subject, and at his confidence in the security of Atlanta by virtue of her altitude."

In the discussion of a question of this kind, the only argument which has any value whatever is that which is comprised by the stern logic of facts. As long as we are utterly ignorant of the true nature and causation of the disease (as we undoubtedly are), theories in regard to its method of propagation are mere idle speculations. To observation alone can we look for conclusions which will be worth considering.

A careful study of the article by our Texan contemporary discloses only two such observations upon which to base his dissent from our proposition, viz.: First, that in 1878 Holly Springs was ravaged by yellow fever; second, that yellow fever has been known to occur (*in a refugee*) on the summit of Lookout Mountain. Hence, "in light of these established facts," our claim that "no city of the altitude of Atlanta has ever been ravaged by the yellow fever" is "morbid sentimentality." Such is the argument about which his remarks center.

We fail to see in the two facts cited any refutation of our position. The fact that Holly Springs suffered from the disease has no bearing upon the question of Atlanta's immunity, since Holly Springs falls very far short of enjoying the altitude of Atlanta. Holly Springs evidently lies below the safety line, and hence it is absurd to draw from her experience for conclusions in regard to Atlanta, which lies above that line. Moreover, Holly Springs is located in the Mississippi valley, only about fifty miles from the Mississippi river, and even though it possesses an accidental elevation, so to speak, its surroundings are those which are characteristic of the river basin in which it is located. We admit that it is not immediately on the banks of a river, but on the north, the west and the southeast streams of considerable size flow within less than ten miles of it. Atlanta, on the contrary, is situated on a spur of the Blue Ridge, the so-called "Piedmont escarpment," where every breeze is laden with the healthfulness and purity of the still higher altitudes in which it finds its birth. Atlanta has been repeatedly exposed to yellow fever by the presence of refugees in large numbers, and even of imported cases, in former years, yet no case has ever originated here. She has never quarantined against the disease, but has repeatedly been a "city of refuge" for the suffering, yet she has always come forth unscathed. If generalizations, based upon the experience of the past are ever warranted, the principle which we have stated in regard to the immunity of Atlanta is fully justified.

We do not claim this immunity for Atlanta alone. Our claim is not a "boast" prompted by local vanity, as our esteemed contemporary implies. Since our former editorial upon the subject was written, a most striking corroboration of our views has been furnished by the experience of Hendersonville, a little city located in the mountains of North Carolina. Strong in the courage of her convictions, this brave little community admitted within her borders a train load of several hundred refugees, among whom were three actual cases of the disease within three days after their destination had been reached. Seven other cases occurred among these refugees, the disease having evidently been contracted before leaving Jacksonville. Two deaths occurred. Not a single additional case followed, even among the refugees themselves. The disease was effectually jugulated by the change of atmospheric conditions. Will any intelligent person claim that yellow fever was no "respecter of altitude" in this case? No better conditions could be desired for a crucial test of the principle which we have maintained. The same remarks hold good in regard to the case of the refugee on the summit of Lookout Mountain.

We are accused of "morbid sentimentality" in the views which we hold upon this question. Yet we have endeavored to look at it as we would look at any other scientific question, in the light of plain facts, and of the evidence before us. If there is any sentimentality in the case, it seems to us to lie upon the side of those who ignore such facts, and whose opinions are born of craven fears rather than of the indisputable evidence before them.

We are further accused of "boasting" of our immunity from the ravages of the terrible scourge. Yet we would say that it is in no spirit of self-glorification that we have referred to this subject. Far be it from us to gloat over our more favorable situation as compared with our sister cities. Atlanta has no selfish ends to gain in throwing wide open her doors to the Florida refugees. On the contrary, an unreasoning selfishness would have prompted a different course. Her charity is untinctured by policy, and inspired by no hope of reward beyond that contained in the promise, "Inasmuch as ye have done it unto one of the least of these, ye have done it unto me."

